# Youth voices and experiences regarding a school-based cognitive behavioral therapy skills intervention: lessons for future engagement and adaptation

**DOI:** 10.1186/s12889-022-14058-z

**Published:** 2022-09-09

**Authors:** Paula Klim-Conforti, Anthony J. Levitt, Amy H. Cheung, Raisa Loureiro, Mark Fefergrad, Ayal Schaffer, Thomas Niederkrotenthaler, Mark Sinyor, Juveria Zaheer

**Affiliations:** 1 Institute of Medical Science, Temerty Faculty of Medicine, University of Toronto, 1 King’s College Circle, Medical Sciences Building, Toronto, Ontario M5S 3H2 Canada; 2 Member of the College of Psychologists of Ontario, Toronto, Ontario Canada; 3 Member of the College of Registered Psychotherapists of Ontario, Toronto, Ontario Canada; 4 Department of Psychiatry, Sunnybrook Health Sciences Centre, Toronto, Canada; 5 Department of Psychiatry, University of Toronto, Toronto, Ontario Canada; 6 Unit Suicide Research and Mental Health Promotion, Department of Social and Preventive Medicine, Centre for Public Health, Medical University of Vienna, Kinderspitalgasse 15, A-1090 Vienna, Austria; 7 Wiener Werkstaette for Suicide Research, Vienna, Austria; 8 Institute for Medical Health Policy Research, Centre for Addiction and Mental Health, Toronto, Ontario Canada

**Keywords:** Suicide, Depression, Anxiety, Universal prevention, School-based, Mental health literacy, Cognitive behavioral therapy

## Abstract

**Background:**

The Cognitive Behavioral Therapy Skills Intervention (or CBTSI) aims to build mental health literacy and knowledge, allowing youth to build resilience and improve mental health broadly. In Ontario, Canada, youth voice is scant and European studies have largely reported on youth factors supporting stigma reduction, help-seeking intentions and overall satisfaction with a given intervention. Process evaluations and implementation that underpin what youth require to embrace mental health literacy interventions, particularly those that embed key learning principles in the everyday curriculum, have not been broached. The goal of this study is to understand both barriers and facilitators to engagement with the CBTSI (an intervention novel in itself because of the combined mental health plus cognitive behavior therapy (CBT) skills principles embedded in literacy) and the resources and structures that students report requiring, to fully engage with such an intervention.

**Methods:**

Student focus groups were conducted utilizing qualitative interviews that were analyzed thematically. Analysis was informed using principles of pragmatism and analyzed inductively using thematic analysis (Braun and Clarke, Qual Res Psychol 3:77–101, 2006), first looking at the whole and then coding for themes, within an interpretivist framework. Youth were in middle school (grade 7 and 8) in Toronto, Canada who had received the CBTSI. Face to face interview guides with iterative questioning were conducted in February of 2020, and these interviews were audio-recorded and professionally transcribed. Teachers randomly chose a subset of youth whose parents consented to the research to ensure ethno-racial similarity to classroom demographics.

**Results:**

There were eight groups with sixty students who participated. Students were 12 to 14 years of age. Major themes were identified: maximizing the opportunities for involvement and self-determination created an atmosphere where confidence and self-compassion could flourish, signalling to the students that they understood and were able to deploy the strategies they were taught; students expressed that the intervention needs to be adapted to enhance personal dignity, respecting both individual wishes and goals in light of the variability in student reported mental health. A model explains the structures and adaptations required to maximize learning based on youth feedback.

**Interpretation:**

Mental health literacy incorporating CBT is a promising population-based health promotion intervention. Future adaptations and implementation decisions regarding the CBTSI need to address the wishes and experiences of these youth. Youth voice in this study explored factors that prevent and promote the uptake of the key lessons within the context of existing variability in student mental health that is often found within the context of a regular classroom. The results should be used to adapt the CBTSI as it is disseminated more broadly.

Nearly half of all mental disorders start by 14 years of age [1]. Mental health difficulties are cited as the number one barrier to educational attainment, often leading to school dropout [2]. Anxiety and mood disorders impair student achievement, impact daily functioning, place students at risk for suicide, and impair social interactions [3, 4]. Improving child and adolescent mental health can profoundly affect life trajectories [5]. While there is broad agreement about the importance of healthy mental development in children and youth, existing service models often lead to suboptimal care for young people [6, 7]. An estimated 20-25% of children and youth identified with a mental disorder receive mental health care in our current mental health care system in Ontario, Canada [8–11] with an estimated 35-60% of the total child and youth population in the United States and Canada who exclusively receive mental health services in school [12, 13].

Universal school-based prevention is one way to reach more youth earlier and may be a means of addressing service delivery challenges when the mental health of youth who are identified require more specialized care and navigation. Universal interventions can improve mental health literacy [14–16], identify persons at risk for suicide [17–20] and anxiety and mood disorders [1, 21, 22]. These challenges have been identified within Ontario and internationally as the highest priority student mental health issues [23–25].

Despite the potential of universal school-based prevention to address youth mental health needs, evidence regarding how students engage with, and experience universal interventions for the purposes of identifying barriers and facilitators to engagement and assisting implementation is lacking. Jorm coined the idea of mental health literacy (MHL), with universal population based application, with the intent to educate the public on matters of mental health and wellness, and what might constitute the need to seek further expert advice, so that one can receive early intervention with the hopes of altering the trajectory of mental illness; much like educating the public about the benefits of adhering to a healthy, balanced lifestyle, with physical exercise, and how it alters the prognostic course of medical diseases [26]. In addition, first aid skills to support others affected by mental health problems is central to MHL [26]. Youth engagement is critical to all mental health services as these services are increasingly shifting away from traditional healthcare delivery venues and into locations such as schools to improve access to varied care [5, 6, 13].

School MHL interventions as a universal preventative tool are increasingly being applied outside of the country [27–29]. There is limited feedback from youth concerning the adaptations that might be required to embrace such prevention programs. Naturalistic settings pose considerable challenges; for this reason, study designs have begun including implementation and process evaluations, informed by qualitative interviews of satisfaction [30]. In Ontario, Canada, youth voice is scant and European studies have largely reported on youth factors supporting stigma reduction, help-seeking intentions and overall satisfaction with a given intervention [31, 32]. Process evaluations and implementation that underpin what youth require to embrace such mental health literacy interventions, particularly those that embed key learning principals in everyday curriculum has not been broached. Taken together, generalizability of mental health literacy interventions, while constructed to support the needs of many, at the cost of a few, may need to be further adapted depending on both the prevalence rates of mental health problems in any given classroom or school and the country, state, or province where the intervention was designed due to varying educational requirements and ministerial emphasis.

This paper draws on qualitative data collected as part of the process evaluation within a randomized controlled trial (RCT) of the Cognitive Behavioural Therapy Skills Intervention (CBTSI). Details of the intervention have been previously published [15] but, in short, it involves imparting CBT skills to middle schoolers while reading ‘Harry Potter and the Prisoner of Azkaban’ in English class. While our RCT study revealed how the intervention impacted these variables, prior publications did not examine how students interacted with and received the curriculum. While our intervention was co-developed with youth from the initial design stages, this qualitative study centers the students’ experience within the context of the delivery of the intervention in a school environment. Realistic approaches to program evaluation move beyond theoretically driven mechanisms through which interventions produce positive change and acknowledge the interaction with context and the dynamic and complex nature of social systems. Realism coupled with pragmatism aims to answers to real world problems (in this case, acceptance of, and optimal learning conditions to support, MHL interventions) within the context of this dynamic social interaction. Theories underpinning the design of the CBTSI include bidirectional social learning, combined with what we know of how kids learn best – through stories-, with Cognitive Behaviour Therapy (CBT), and by imparting stories of resilience and coping through an engaging narrative, we wondered if school implementation of this intervention could augment suicide prevention efforts.

The study aimed to explore students’ experiences of the intervention, to better understand barriers and facilitators to engagement, perspectives on the lesson planning, and ultimately to incorporate their feedback to improve engagement. Process evaluations and implementation that underpin what youth require to embrace such mental health literacy interventions, particularly those that embed key learning principals in everyday curriculum has not been broached. A model is suggested which illustrates the component requirements of school-based mental health literacy (MHL) intervention implementation incorporating CBT (MHL + CBT) to inform the resources and structures that students report requiring, to fully engage with such an intervention. This augments and adds vital information for consideration before widespread dissemination in urban, multicultural, diverse school boards.

## Intervention

The program that is being evaluated is a 3-month, teacher-led, CBT skills intervention to provide grade 7 and 8 students with coping skills to regulate their emotions which in turn is intended to build resilience and distress tolerance. While reading “Harry Potter and the Prisoner of Azkaban,” students engage in discussion about key learning objectives such as identifying risk and protective factors, basic cognitive restructuring techniques, behavioural interventions to improve mood, and promoting help-seeking behaviour should these skills fail to improve distress tolerance. Students learn to recognize how depression and anxiety manifest in the characters in the novel and additional exercises augment student learning through a discussion of the symptoms, behaviors and thoughts to recognize depression and anxiety in themselves and others. The intervention teaches key concepts in a developmentally appropriate format. Students can maintain private workbooks if they desire and can share with classmates, teachers, or parents as much or as little as they are personally comfortable. Participating teachers will be provided with a manual outlining standardized key learning objectives. Core lessons are mandatory, with each chapter containing a key learning objective, to be taught. Additional lessons meeting language arts requirements will be created by teachers, permitting local curriculum needs to be met. The intervention will be implemented as a universal or tier-one school system support model, emphasizing healthy responses to distress. Suicide will not be mentioned in the intervention, apart from a brief note that suicidal ideation can be a symptom of depression. Teacher training will be intentionally kept brief to permit differentiated instruction within the classroom. Differentiated instruction accommodates or modifies the learning experience to meet the needs of students who learn differently. Differentiated instruction can involve adjusting content (for example, media to deliver content and instructions), processes (exercises and practices students perform to understand the content better) and products (tests and projects that demonstrate student understanding) [33]. Fidelity checks to ensure learning objectives will be gleamed from reviewing homework assignments and checklists.

## Reflexivity statement

Paula Klim-Conforti, who conducted the focus group interviews, is a Registered Member of the College of Psychologists of Ontario and a graduate student in the Faculty of Medicine, Institute of Medical Sciences program at the University of Toronto. She has been registered for 20 years, for 15 of which she was employed by the school board where this qualitative study was conducted. The focus of inquiry was on obtaining helpful answers to practical questions.

## Methods

### Theoretical framework

This study draws on programme evaluation with a pragmatic approach. The main author identifies as a mixed-methods researcher who weighed the delicate balance between several quantitative researchers and one qualitative researcher. For the lead author, the importance is on the research question(s). Pragmatists emphasize practical questions in search of useful and actionable answers based on real world constraints of limited time and resources [34]. Creswell [35] adds that mixed methods researchers use pragmatism to permit the exchange of ideas without allegiance to a particular epistemological or philosophical and theoretical position. The quite concrete and practical questions that people can envision to make the world a better place and discussion of what is working can be addressed without such constructs. From a pragmatic perspective, the designs, and methods for collecting and analyzing data are selected based on the stated research goals and guided by a researcher’s personal values [36, 37]. We draw on Shenton’s model of credibility, transferability, dependability, and confirmability to establish rigour and trustworthiness as “practical” implies a basis in one’s experience of what does and does not work [34, 37]. Data collection steps that established trustworthiness involved “on the spot” member checks, negative case analysis, debriefing sessions with the qualitative expert on the research team (JZ), use of reflective commentary, peer checking, thick descriptions, and an audit [37]. The qualitative expert theoretical perspectives informing interview reflections and debriefing advice included that reality is socially, intersubjectively, and experientially created (“relativist ontology”) [26]. Each individual’s understanding of the world is central to and influenced by their understanding of themselves and others (“subjectivist epistemology”) [26]. Investigators and participants are connected: as the inquiry proceeded, investigators and participants co-created findings and knowledge through dialogue [26].

Reflective questioning occurred with a tolerance for ambiguity to be receptive to the co-creation of emergent categories [38, 39], which were iteratively refined to capture the classroom interactions.

### Data sources and study design

The current study is a qualitative exploration complementing our prior RCT results [15]. The research was introduced by study staff first at the school level and then by the teachers within each classroom. We explored with the students who participated in the focus groups if these research introductions set the stage for either the acceptance of, or rejection of, the intervention. Students described their experiences of how each of the classroom lessons was broached by the teachers. The rollout of the research and intervention lessons was to inform what research protocols and curriculum content were working well and what aspects might require revisions. The overall purpose was to understand the extent to which participants perceived the intervention to be useful or not, to inform intervention development and dissemination, and to offer insights into classroom interactions, which established themes that formed a model of MHL + CBT universal intervention implementation within school boards. Collectively, these themes and the resulting model improve intervention adoption and facilitate widespread dissemination.

### Sample interview questions and topics

Open Exploration: The main purpose of this part of the interview is to allow the participants to express themselves as freely as possible. The participant decides what is important to him/her, so let them talk about whatever they want to as much as possible. That means we do not control the agenda rigidly but try to allow maximum narrative space.

Examples of Open-ended questions for open exploration:

- You can start with whatever you want to talk about first (if participants asked what they should start with).
- What did you think when you heard that you would be learning about HP at school? What did you think about learning mental health through Harry Potter?
- Tell me about one of the lessons you had. What were the parts of the unit that stood out?
- What about the program, did you like? What didn’t you like? Did you forward to it? Were you surprised by it?
- Has the program changed the way you think about yourself? What about other people?
- Would there be anything that you would change?

Structured Inquiry: The purpose of structured inquiry is to focus on specific areas or issues we are interested in but have not been addressed by the participants in the Open Exploration section.

Focused exploration topic and question examples include:

Mental health literacy

       • What did know about mental health before the unit? What do you know now?
       • In the Unit we learned about anxiety, sadness, and distress, and we also learned about “stress busters”. What did you think about all that? Did any of you use the learning in your life? If not, how do you think got in the way?
            ◦ How do you think it can help you with? How do you think it is not helpful?
       • How would you describe depression, anxiety and distress to a friend?
       • Did the unit change the way you think about stressful situations? Can you give me an example?
       • What did you learn about thinking errors people can have when depressed, anxious or in distress?
            ◦ How can you recognise when you or someone else is having a distorted thought?

Communicating about one’s feelings

- Sometimes it can be hard to talk about our feelings. When you are feeling stressed, who do you talk to?
- What gets in the way of talking to people? Have your thoughts about this changed?

Planning the Program

- Do you think the idea of having a mental health unit like this in English class is a good idea?
- Would you recommend it to other classes and schools?
- Is there anything else you’d like us to know?

### Procedures

Study investigators approached school system administrators to recruit English teachers across an urban, diverse school board in Toronto, Ontario. Eligible participants were students who were 12 to 14 years of age or in grades 7 or 8, and their teachers who received training in delivering the intervention and agreed to do so in the 2019-2020 academic year. All students in each participating class received the intervention, and only those whose parents provided explicit consent for the research component were included in the study. The intervention was designed to teach skills that would be beneficial for all students, and as such, the intervention was part of the regular language arts curriculum.

### Data collection

Student focus groups were selected based on a convenience sample with some purposeful sampling, ensuring that each group was demographically representative of a typical classroom in each participating school. There were 6-8 students involved in each of the 8 groups with 60 student participants in total. We did not capture demographic data on participants as we did not have ethics approval to do so. Teachers randomly chose a subset of youth whose parents consented to the research to ensure ethno-racial similarity to classroom demographics. Face-to-face 60- minute focus group interviews at the schools using interview guides with iterative questioning were conducted in February 2020 by PKC, or within days of intervention completion, and these interviews were audio-recorded and professionally transcribed. Additional notes to capture non-verbal information were made after each focus group. Transcripts were not returned for participant comment or correction due to the global pandemic, and ethics approval did not permit identification for future telephone inquiries. However, member checks took place “on the spot.” Only the principal investigator (PKC) and participants were present during interviews. Data collected across all interviews were interpreted by the lead author (PKC) using thematic analysis [40], and several transcripts were reviewed and discussed with the senior qualitative methods expert (JZ). The interview guide was co-developed by PKC, JZ and RL.

### Analysis

**Fig. 1:**
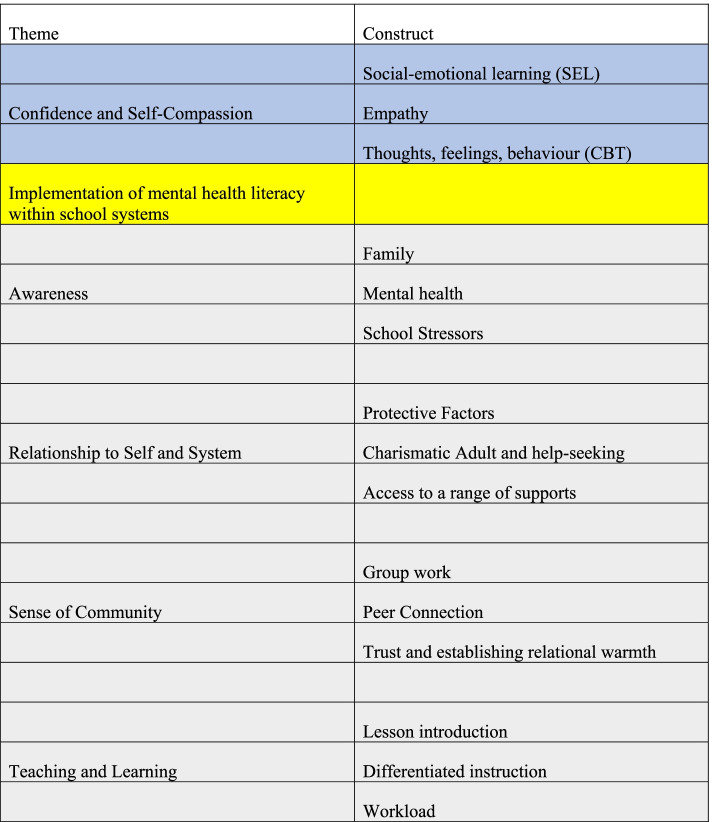
The core theme and corresponding categories in blue signaled for the students that the learned skills and strategies were effective and could be applied themselves, improving self-awareness. Sub-themes and corresponding categories below the yellow line are requirements for successful school based implementation of the MHL + CBT intervention

Qualitative data was processed using principles of pragmatism and analyzed inductively (working back and forth between the general and specific to solve a problem), first looking at the whole and then coding for themes [39, 41]. Each transcript was read, coded, re-read, and recoded as necessary by PKC with feedback, review, and discussion from JZ [40]. Codes were grouped into themes which created a thematic map. Themes were refined and organized (Fig. 1).

We aimed to define and construct an account of the exploratory objectives using constant comparison grounded in study data within a naturalistic setting following Braun and Clarke’s [40] description of the analytic process. We recognize how social contexts, interactions, sharing viewpoints and interpretive analysis of the students and the researcher(s) influence understanding [38, 42].

Nvivo 12 was used to identify passages with keywords and extract coded passages for further synthesis and identification of themes. The unit of analysis for program evaluation uses the mention of the overarching lessons and user experience with it. Themes were identified from the data with constant comparative reflection (Fig. 1). This study met 21 of the 21 Standards for Reporting Qualitative research.

### Ethics

The Research Ethics Board approved this study at Sunnybrook Health Sciences Centre (Project Identification Number: 238- 2018) and the Toronto District School Board’s Research and Development Department. A minimum of 2 weeks was provided to permit parents to provide written consent. Consent was obtained at the beginning of the multimethod study. At the commencement of the focus groups, students were engaged in a general discussion to ensure that the transcriber, who was external to this research group, could identify each of the voices. This general discussion assisted the coder in understanding each of the participants’ flow of thought. Positioning the student in the room helped to understand any dynamic interaction that may have taken place. Instructions were provided to participants to avoid using any identifiable information, and they were reminded of the consent to audio record. Should any identifiers have been accidentally recorded, this information was deleted from the recording while transcribed. Following hard copies and electronic transcription of the groups, the recordings were destroyed. In short, there was no way to identify focus group participants, teachers, schools, or classrooms.

## Results

Five themes and fifteen associated constructs were identified from the student data (Fig. 1).

Students perceived the MHL + CBT intervention to be successful if *confidence and self-compassion* were developed. Confidence and self-compassion were achieved by collaboratively navigating emotions and developing empathy, which resulted from becoming more attuned to and understanding how thoughts, feelings and behaviours interact with and affect one’s responses to situations. Overall, increased self-awareness, establishing strong relationships between the students and the school system, fostering a sense of community and teaching and learning were themes that interacted with student confidence and, therefore, the successful implementation of the school based MHL + CBT intervention. Linking the themes together through the co-construction of knowledge produced a model to improve the implementation of a school based MHL + CBT intervention.

### Confidence and self-compassion

Students discussed their experiences interacting with the facilitators, which either enhanced or reduced their positive perceptions and experiences interacting with the intervention. There seemed to be relational dependence that resulted from the dynamic interaction with the facilitators and the delivery of the content which resulted in students experiencing improved confidence and having self-compassion or not. The development of confidence and self-compassion signaled for the students that the learned skills and strategies were effective and could be deployed as needed and applied by themselves. Barriers were often in keeping with the themes and constructs discerned but framed in the opposite way to permit further reflective comparison. For example, if the facilitator was not encouraging and inviting of conversation, students felt uncomfortable, which eroded both their participation and confidence.

P1“After she (the teacher) was done talking, we, after a lot of people in our class started raising their hands, and then she said it was all done, you couldn't really tell someone else how you felt about it, because you felt, kind of, embarrassed to. And you didn't really want to raise your hand and say something.”

On the other hand, if the facilitator demonstrated both a clear and comfortable willingness to engage with the students and encouraged them to set the tone and take the lead in class discussions, students’ felt validated. This validation improved their confidence and self-compassion and as a result, youth were more willing to engage in the lessons or group.

P2 “This program was really good for, like, giving good advice, because it’s like, for me, or I feel like, I feel like for most kids, it’s like, their way of coping with all this negativity, this energy, is to be, like, having like a confident mentality…..like, oh I am better than this, like, I’m confident, I’m happy, like, I’m better than, I’m strong….the tips here are filled with compassion.”

Students identified social-emotional learning, empathy and cognitive behavior therapy as catalysts that increased their confidence and self-compassion.

Students described how deliberate and persistent they were in categorizing the thoughts, feelings, and behaviours [Cognitive Behavioral Therapy (CBT)] of the characters in the novel and applying these lessons to themselves.

P3 “So he came, he overcame his fear of dementors with the help of Lupin. And he, he was, he gets, like she said, he gets traumatized every time he sees a dementor, of his mom’s screams and stuff. So, he overcame it and had the confidence to. I think he (had) the confidence to do that because of his friends, they made him not be afraid so next time he fights the dementor, he would succeed instead of going into a seizure.”

Students navigated, what at times were, very abstract and not clearly defined social-emotional reactions [Social-emotional learning (SEL)] whether delivered through the lessons or experienced during interactional opportunities provided within the classroom situation. The lessons and opportunities resulted in increased empathetic reactions (Empathy). There were many individual and interactive verbatim quotes such as the examples below that pervaded many of the student responses. Students seemed to benefit from the CBT novel examples provided by their peers, which they revisited in self-reflection. This helped students develop an appreciation for different perspectives and enhanced student empathy.

P4“When we had to reflect on what strategies like CBT to learn to cope with, say, depression, and you were comfortable sharing, you would see others starting to share too and then you noticed someone had a different error in thinking than you, and let’s say your perspective was not working, you would try theirs and we learned everyone sees things differently, reacts differently and works differently.”

By applying SEL students recognized the continuum of mental health (wellness to higher clinical needs) and were able to apply mental health literacy to their own social-emotional functioning.

P5 Wellness: “When I started reading the book, it was like he (Harry Potter) has stress like me. But my stress is different from his stress. It was good (to realize that) because people will learn we all have to deal with different things, and we all don’t have the same troubles.”

P2 Youth endorsed Clinical symptoms [part of a 3-way conversation (Researcher, 2 students); on the spot check]:

“When people are depressed, like…. people, like, seem to have this expectation, you can do this, you can do that, and like, sometimes it’s too much, the weight of it becomes, like, overbearing and it comes to a point where you can’t do anything about it, and people think you are okay with it.”

P10. “Um, I have depression, I’ve been through depression a lot of times, like I feel like when Harry started opening up and Harry, then his friends helped him, and that’s, like, from that part, I realized, a lot of my friends are also helping me get through my depression…..I felt (realized) like people were trying to open up to me but I just didn’t, I just couldn’t understand what they were saying (at the time).”

Students who are aware of their own mental health needs were able to develop self-compassion. Those who were able to develop self-compassion seemed to elicit empathetic understanding with one another. As two students commented:

P6“I deal with a lot of mental health issues and when I was, when I started reading Harry Potter, it’s kind of just took me to a different world where I didn’t have to worry about my problems, and I could approach my problems later with a new lens.”

P7 “I have more empathy for people now because you never know what they are going through. It made me treat people with more respect, just by looking at them, and talking to them a little, made me realize how they might be feeling.”

### Awareness

Students described an increased awareness of themselves and others in accordance with a better understanding of mental health and clinical needs, finding a balance between asserting themselves as individuals and when family involvement or support was required. These learned skills when trying to manage stressors made more students aware of when to seek help. Many students felt that being able to reflect on and/or have a discussion of these constructs either in class, or with another adult, led to enhanced awareness. Mental health literacy seemed to ground perspective taking towards’ oneself and others.

P8 “Oh yeah. Mental health in schools is a very big issue and by doing this unit you can tell that everyone has at least a bit of mental health (challenges) that they address.”

P9 “Um, like, how she said, like, before this unit, I see the series and the movies, like Harry being brave and courageous, or Hermione being smart and Ron being kind. But now, I’ve seen this mental health added to this, I noticed that there’s a lot more, a lot more things included into the characters that are different.”

Students expressed a distinct desire to seek help where they were most comfortable. This acknowledgement demonstrates a sound understanding of the lesson on protective factors.

P2 “Yeah it was great because I do not even talk (others expressed they do) to my parents about this, I just only talk with my friends…….it actually feels good to talk finally …. because you never know what someone is going through.”

### Relationship to self and system

Students described varying views of their *relationship* with adults at school and did not understand what system-level support (both internal and external) could be available to them. They expressed ongoing support increased the likelihood of mastering distress tolerance because of the relationships that would be formed, which they noted, again, was a protective factor.

“I feel [like] there are students who have no one at home that they feel comfortable going to, parents or siblings or anything ……they don’t have anybody, and sometimes at school too, so having a designated guidance counsellor or professional would be good like protective to have.”

Others expressed having a clear support plan would have been helpful. The importance of relationships could not be over emphasized.

P2“It would be easier to open up to a professional because you won’t feel judged.”

P11“The students and teachers. They don’t talk to each other…they’re not close to them. Like they could say, oh you can talk to me anytime, it’s confidential and everything and you guys can go to your teachers and tell them. It’s not true….it depends on how safe of a space teachers provide.”

P10“Let’s say we don’t feel safe enough to actually talk to like our peers or our friends, I feel like there needs to be a safe space just between, like a trusted adult, to have a talk with, just to let someone know what you’re going through.”

### Sense of community

The establishment of a sense of community whereby peers interacted during group work established an increased sense of cohesion in the classroom and being permitted to have the time to discuss lessons as they were introduced fostered trust. The following quote seemed to summarize this well.

“(We completed the Harry Potter intervention) at the beginning of the year and not everyone knew each other or were really close, I guess you could say, but because we often talked in groups, in the beginning I wasn’t really going to share anything, and I might have been uncomfortable, but it definitely brought us together. I was able to express myself (as the intervention moved along) and now I trust them more.”

A student expressed how the curriculum was received when community and classroom tone did not set the foundation for mental health exploration.

P12 “School never talks about it (mental health), you can’t bring it up one time and expect us to talk about it. I just do not trust them.”

### Teaching and learning

How the research, intervention and lessons were introduced set the tone for how receptive students were to learn and interact with the material, which was associated with teacher comfort level in teaching the concepts. Providing the students with a balanced workload and differentiating instructions additionally facilitated engagement and learning.

P13“Our class thought this was random because it was different from what we usually learn at school…. since we learned about stressbusters and stuff, our class didn’t really relate to it…it is important that we learn about stressbusters and stuff, but it wasn’t introduced in a way that would make us want to learn about it….it just came out of no-where.”

Extending lessons and scaffolding key learning objectives using principles of differential instruction would have facilitated understanding.

P14“If we had chosen a book like more at our reading level and maybe (discussed) real world things that we can relate to and talk about, it would have been easier to understand.”

Balancing academic demands and key learning objectives through ongoing discussion of class requirements and student load-maintained engagement (or not).

P15“I think near the end it got stressful, because other classes didn’t have this work, so it was an add on to what we were doing and, while it was interesting, and we learned a lot, the load was hard…. it’s like don’t have stress (or find healthy ways to manage stress) but someone’s giving us more stress.”

**Fig. 2:**
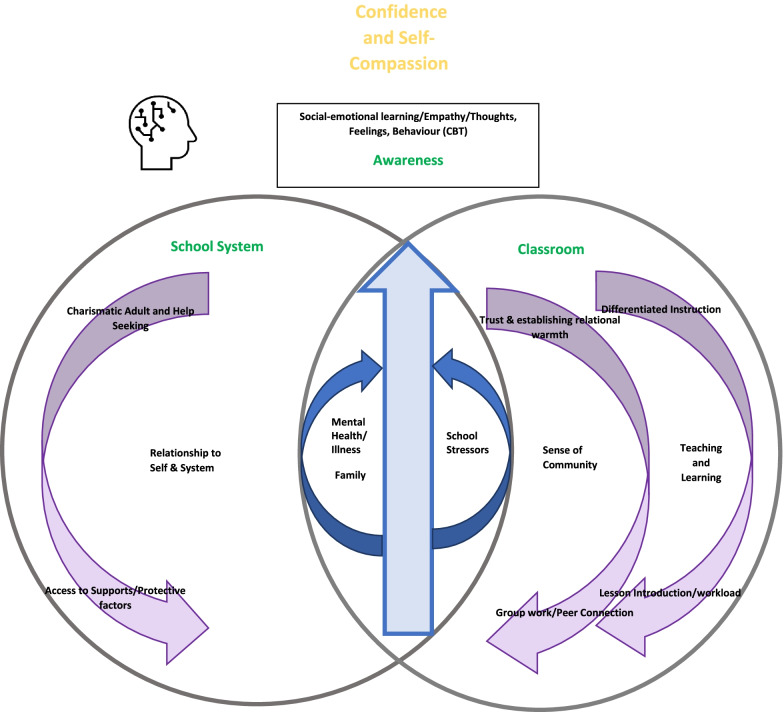
Mental Health Literacy Implementation Model

Linking the themes together through the co-construction of knowledge produced what the students described as helpful insights into what they considered to be the component requirement for a universal MHL + CBT intervention that the youth will accept and find useful (barriers and facilitators to engagement: Table 1). We developed a model to understand how universal methods derived from MHL can be improved to assist adoption by a school board (Fig. 2). The model depicts the dimensions of school-based MHL + CBT implementation and the relations to one another with the Harry Potter Cognitive Behavior Therapy skills intervention at the foundation.

**Table 1:** Examples of barriers and facilitators to engagement

Facilitators	Barriers
Attitude of the Moderator: 1. A visibly clear and comfortable willingness to engage with students about mental health.	Attitude of the Moderator: Not encouraging nor inviting of conversation.
2. Permitting space for students to “take the lead” discussing key lessons.	Lack of direct instruction for students (when required) linking emotion-action-thought.
Relationship to the school: 1. An identifiable adult who can further discussions and/or referrals as needed. This need not be the teacher.	Relationship to the school: School and/or teacher not previously known by students to have facilitated or participated in mental health initiatives.
2. Having established trust with a known identifiable adult.	Student’s feeling not understood and accepted.
Student knowledge: 1. Knowledge of individual protective factors.	Character Identification Having choice in literacy novel.
2. Upfront discussion and understanding of system level support both internal and external to the school along with clear support plan of action.	Incorporating or having culturally relevant examples and scenarios in the story.
Process: 1. Group work and peer interactions.	Process: For concepts that were determined by peers to not often be discussed, ample opportunity to have questions answered.
2. Permitting space for CBT practice.	Inability to balance workload and differentiate instruction.
3. Student involvement in the implementation process.

## Discussion

The purpose of this study was to explore students’ experiences of the CBTSI, to better understand barriers and facilitators to engagement, perspectives on the lesson planning and content, and to ultimately incorporate their feedback to improve engagement. Facilitators with previous school involvement in mental health initiatives, facilitators or interventionists who could be agile and adaptable to students’ needs, enabled engagement. Additional enablers were a) being comfortable with the material, b) knowing when to refer on, c) being prepared to differentiate instruction (by using visual, audio, linguistic and kinesthetic pedagogical approaches), and c) to differentiate the end product (by way of dividing students based on mastery of content), taken together, improved mastery. Barriers were framed the opposite way; for example, the lack of previous mental health intervention history in a given school was a barrier to engagement.

Students perceived the MHL + CBT intervention to be successful if *confidence and self-compassion* were developed. Confidence and self-compassion were achieved by collaboratively navigating emotions and developing empathy, which resulted from becoming more attuned to and understanding how thoughts, feelings and behaviours interact with and affect one’s responses to situations. Overall, increased self-awareness, establishing strong relationships between the students and the school system, fostering a sense of community and teaching and learning were themes that interacted with student confidence and, therefore, the successful implementation of the school-based MHL + CBT intervention. Despite the increasing use of universal interventions, there is limited empirical evidence to indicate how students respond to and engage with them. From this, we could develop a model to understand how universal methods derived from mental health literacy can be improved to assist in implementation. This augments and adds vital information for consideration before widespread dissemination in multicultural, diverse school boards.

A proportion of students within the focus group self-report a higher burden of mental health symptoms and/or confirmed clinical presentations, some with and without a clear pathway to care. Depending on the student’s professional and/or mental health self-assessment, students felt supported by an array of ‘adults.’ That is, their peers, the teachers, or professionals (if available). If student needs matched the adults’ “training,” students flourished and maximized the benefits of the intervention. This supports and signals the need to have various educational and training needs met and tailored within any given school within a school system, particularly when CBT components are added. While the CBT components of our intervention are relatively basic, as expected, the students had a broad range of familiarity with them (limited to well versed). Students viewed the development of self-confidence and compassion (the primary theme) as pivotal to whether the intervention was successful. Factors that interacted with developing these traits were proportional to the degree to which the student and, reportedly, the teacher felt equipped to intervene [43].

More specifically, based on our data, students perceived themselves to be more confident and self-compassionate if facilitators encouraged and invited them to the conversation. This means facilitators need to both permit practice of CBT skills and guide self-awareness through social-emotional learning via the characters in the novel and/or student/individual examples. This created the space for perspective taking and empathic responding, sometimes not achieved until facilitated with the interventionist. We heard essential distinctions in how students framed their actions and interactions as responses within specific contexts. They were embarrassed when no follow-up was broached and validated when they could express themselves free of censure. Self-compassion signaled for the students that they learned to be less self-critical, defined as the tendency to set unrealistically high self-standards and excessively scrutinize one’s behaviors at the expense of adaptive coping mechanisms [44, 45]. Self-compassion can be viewed as the end product of appropriate cognitive appraisal, examined during the CBT exercises, a core component of the intervention. Improperly attributed cognitive appraisals are a significant risk factor for a host of psychopathologies [46], including suicidal thoughts, behaviors, and related risk factors (depression, anxiety) [47].

Students who self-disclosed higher clinical mental health needs (anxiety and/or depressive symptoms or diagnosis) expressed the desire to have access to professionals in the school environment and saw this as crucial following self-exploration and conveyed that professional access was a necessary protective factor. Consistent with the literature, students wanted various mental health services in their schools and indicated this was an important factor in accessing care [5, 6].

Our review of school-based interventions indicates that mental health literacy interventions are promising. MHL interventions have been postulated to be an essential determinant of help-seeking as they are among the few interventions found to be significantly better than controls [16, 18, 19]. Our findings support thatstudent help-seeking depends on the relationships they formed with a trusting adult(s) and between the student and the school system. In contrast, prior evidence regarding the ability of universal school-based interventions to reduce depression and anxiety is mixed [1, 18, 21, 22, 48]. Students participating in our focus groups broadly represented the diversity captured in any given classroom. Teachers randomly chose a subset of youth whose parents consented to the research to ensure ethnic and racial similarity to classroom demographics. Groups seemed to represent the continuum of mental health needs often described in more traditional clinical settings. It is postulated that evidence supporting school-based interventions is mixed because of the few (if any) studies incorporating multi-level interventions and/or analyses within prevention designs [5].

There remains disagreement on the focus and design of school-based interventions. This is due to a variety of factors such as conflicting evidence justifying dissemination of such interventions [49, 50], differences in research design and conceptualization, making comparison challenging [1]; few opportunities to conduct rigorous studies in schools systems [51–53], varying stakeholder buy-in [6, 22], and a general lack of, and competing understanding of, advances made within the school systems to meet these challenges [54, 55]. Streamlining and bridging a clear pathway to support from within and outside the school system for those students who do not fall under the “transition” age warrants investigation so students are not left behind [6].

Students’ perception of the intervention was strongly influenced by how the intervention was introduced and the school’s previous initiatives regarding mental health. Specifically, the ability of the facilitators to be at ease with the topics and lessons, the overall quality of the interactions, and the school’s culture influenced students’ perception and receptivity. The cultivation of these fostered trust and were distinct to the various school sites. For example, students would distrust the process if the intervention were reinforced/re-introduced as a regular classroom language arts activity without context. In contrast, if time was taken to reinforce why the program is being delivered and facilitators outlined the structure and promoted open dialogue, students felt comfortable engaging more with the process. If the school had not undertaken such mental health initiatives in the past, students would have been more cautious about proceeding. This cautiousness could range between argumentativeness and quietness (depending on individual character and perhaps class culture).

In addition, student interactions with other peers, the teacher and/or adults in the school and how they responded to student complexity of needs and variations needed in their mental health care appeared to foster help-seeking. Students became more aware of protective factors and actively sought solutions and connections.

Responding to the varying complexity of student mental health needs was crucial. However, it was equally crucial to adapt the language or curriculum lessons, which complimented the intervention, to the varying academic levels in the classroom. While some students required concrete and tangible “take-aways,” being challenged to infer the characters’ emotions or identify emotions in themselves and others fostered inclusive and mutually supportive interactions. Some students requested that the key learning objectives and CBT principles be transferred to books with more concrete representations of emotion-action-thought and reduced inference and abstraction; other students enjoyed the multiple perspectives their peers and teachers shared, challenging them to extend their thinking. Students varied in their ability to manage the workload and “deliverables;” that is, students remained engaged if the marked work included written, oral, and visual submissions.

Our overall model was derived from the needs expressed by youth; that is, the model is youth-centred, with students’ needs informing the types and mixes of intervention design and services provided. The intervention needs to be adaptable to enhance the personal dignity of youth, respecting their wishes and individual goals and involving them in the process. This promotes an atmosphere where confidence and self-compassion can thrive. Specifically, this study highlights the use of universal interventions and how they are embraced, which is an evolving process developed through fostering productive and predictable interactions and relationships. These interactions and relationships underpin the need for clear paths to varying levels of support for students, facilitators (the teachers) and schools.

### Limitations

The main limitations to the study were a) not having ethical approval for demographic information, which also impeded understanding of underrepresented groups such as LGBTQ2+ and indigenous students; and b) recognizing that the researchers’ position and perspectives inevitably influence access to findings and knowledge construction. These limitations were mitigated by adopting pragmatism and a thematic analysis [40] approach in keeping with an interpretivist framework, which asserted research rigour. Added to these, although Shenton’s model [35, 38] of trustworthiness was followed due to PKC’s background, the data was reviewed and discussed by only one senior qualitative research expert on our team, which positivists can question. Strategies to improve rigour included contrasting student accounts within and across cases, “on the spot” checks for confirmation, and establishing researcher reflexivity through memo writing and questioning one’s preconceived notions while constructing the model and determining the barriers and facilitators. The research was cut short due to the pandemic, making re-engaging with the participants impossible due to confidentiality requirements.

Understanding the school system in which such a study is being implemented, or partnering with a range of professionals who currently, or could, service the schools implementing such a design, may circumvent many challenges often associated with more complex research designs. Future research may include commencing with a focus group on multiple levels, with system leaders, professional staff, educators, and students to assist with research design planning. In addition, particular attention might be paid to including underrepresented groups such as LGBTQ2+ and indigenous students. While our study included students representative of the diversity in each of the classrooms approached, we did not collect demographics. Future research should consider follow-up with students and the longer-term impacts, that is, the importance of applying learnt skills.

## Conclusions

This study provides an understanding of barriers and facilitators to engagement, perspectives on lesson planning, and, with this information, future implementers of universal, school-based mental health interventions can ultimately incorporate their feedback to improve engagement. The model identified core constructs underpinning what students perceive as successful mental health implementation of a universal intervention. Our findings indicate that the ability of facilitators to be at ease with the topics and lessons, the overall quality of the interactions, and the school’s culture were crucial to facilitating the successful delivery of this intervention. Viewing students as responsive and agentic, rather than passive recipients of learning requirements, as well as accounting for the complexities of their academic and mental health needs, fostered trust, relationships, and help-seeking. While we cannot account for how many students in total may have sought help or how many were already receiving support, we know that several students located PKC while she was in the schools to support them in actively seeking help. This model needs further testing, validation, and development in different school board contexts. The model and facilitators and barriers identified contribute to the literature by outlining the resources and structures that students report requiring, to fully engage with such an intervention. This augments and adds vital information for consideration before widespread dissemination in multicultural, diverse school boards.

## Data Availability

n/a. We do not have Research Ethics Board approval to share data and materials.

## References

[1] Caldwell DM, Davies SR, Hetrick SE, Palmer JC, Caro P, López-López JA, Gunnell D, Kidger J, Thomas J, French C, Stockings E, Campbell R, Welton NJ (2019). School-based interventions to prevent anxiety and depression in children and young people: A systematic review and network meta-analysis. Lancet Psychiatry.

[2] Owens M, Stevenson J, Hadwin JA, Norgate R (2012). Anxiety and depression in academic performance: an exploration of the mediating factors of worry and working memory. Sch Psychol Int.

[3] Ruch DA, Sheftall AH, Schlagbaum P, Fontanella CA, Campo JV, Bridge JA. Characteristics and precipitating circumstances of suicide among incarcerated youth. J Am Acad Child Adolescent Psychiatry. 2019;58(5) Available from. 10.1016/j.jaac.2018.07.911. 10.1016/j.jaac.2018.07.911 PMC9721273 30768395

[4] Ruch DA, Heck KM, Sheftall AH, Fontanella CA, Stevens J, Zhu M, Horowitz LM, Campo JV, Bridge JA (2021). Characteristics and Precipitating Circumstances of Suicide Among Children Aged 5 to 11 Years in the United States, 2013-2017. JAMA Network Open.

[5] Fazel M, Kohrt BA (2019). Prevention versus intervention in school mental health. Lancet Psychiatry.

[6] Schwean V, Rodger S (2013). Children first: It’s time to change! Mental health promotion, prevention, and treatment informed by public health, and resiliency approaches. Canadian J Sch Psychol.

[7] Cohen E, Coller RJ (2020). Evaluating Integrated Care for Children: A Clarion Call or a Call for Clarity?. Pediatrics.

[8] Georgiades K, Duncan L, Wang L, Comeau J, Boyle MH (2019). Six-month prevalence of mental disorders and service contacts among children and youth in Ontario: evidence from the 2014 Ontario child health study. Can J Psychiatry.

[9] Mental Health Commission of Canada (2017). Bridging the Gap. Mental Health Commission of Canada.

[10] Lean D, Colucci VA (2013). School based mental health: A framework for intervention.

[11] Richardson T, Stallard P, Velleman S (2010). Computerised cognitive behavioural therapy for the prevention and treatment of depression and anxiety in children and adolescents: A systematic review. Clin Child Fam Psychol Rev.

[12] Ali MM, West K, Teich JL, Lynch S, Mutter R, Dubenitz J (2019). Utilization of mental health services in educational setting by adolescents in the United States. J Sch Health.

[13] Cole E, Kokai M (2021). Consultation and mental health interventions in school settings: A scientist-practitioner’s guide.

[14] Conforti P, Zaheer R, Goldstein BI, Levitt AJ, Schaffer A, Fefergrad M, et al. The feasibility of a Harry potter-based cognitive-behavioral therapy skills curriculum on suicidality and well-being in middle schoolers. Can J Psychiatry. Available from. 2020. 10.1177/0706743720944046. 10.1177/0706743720944046 PMC7564693 32701402

[15] Klim-Conforti P, Zaheer R, Levitt AJ, Cheung AH, Schachar R, Schaffer A, Goldstein BI, Fefergrad M, Niederkrotenthaler T, Sinyor M (2021). The impact of a Harry potter-based cognitive-behavioral therapy skills curriculum on suicidality and well-being in middle schoolers: A randomized controlled trial. J Affect Disord.

[16] Wasserman D, Hoven CW, Wasserman C, Wall M, Eisenberg R, Hadlaczky G, Kelleher I, Sarchiapone M, Apter A, Balazs J, Bobes J, Brunner R, Corcoran P, Cosman D, Guillemin F, Haring C, Losue M, Kaess M, Kahn JP, Keeley H, Musa GJ, Nemes B, Postuvan V, Saiz P, Reiter-Theil S, Varnik A, Varnik P, Carli V (2015). School-based suicide prevention programmes: the SEYLE cluster-randomised, controlled trial. Lancet.

[17] Pistone I, Beckman U, Eriksson E, Lagerlöf H, Sager M (2019). The effects of educational interventions on suicide: A systematic review and meta-analysis. Int J Soc Psychiatry.

[18] Robinson J, Cox G, Malone A, Williamson M, Baldwin G, Fletcher K, O’Brien M (2013). A systematic review of school-based interventions aimed at preventing, treating, and responding to suicide-related behavior in young people. Crisis.

[19] Schilling EA, Lawless M, Buchanan L, Aseltine RH (2014). “Signs of suicide” shows promise as a middle school suicide prevention program. Suicide Life-Threat Behav.

[20] Zalsman G, Hawton K, Wasserman D, van Heeringen K, Arensman E, Sarchiapone M, Carli V, Höschl C, Barzilay R, Balazs J, Purebl G, Kahn JP, Sáiz PA, Lipsicas CB, Bobes J, Cozman D, Hegerl U, Zohar J (2016). Suicide prevention strategies revisited: 10-year systematic review. Lancet Psychiatry.

[21] Moreno-Peral P, Conejo-Cerón S, Rubio-Valera M, Fernández A, Navas-Campaña D, Rodríguez-Morejón A, Motrico E, Rigabert A, de Luna J, Martín-Pérez C, Rodríguez-Bayón A, Ballesta-Rodríguez MI, Luciano JV, Bellón JÁ (2017). Effectiveness of psychological and/or educational interventions in the prevention of anxiety. JAMA Psychiatry.

[22] Joshi A, Bow A, Agius M (2019). Pharmacological therapies in bipolar disorder: a review of current treatment options. Psychiatr Danub.

[23] Live life: preventing suicide, 2018 http://www.who.int/mental_health/suicide-prevention

[24] Mental Health Commission of Canada (2013). School-based mental health in Canada: A final report. Mental Health Commission of Canada.

[25] Kutcher S, Wei Y, McLuckie A, Bullock L (2013). Educator mental health literacy: A programme evaluation of the teacher training education on the Mental Health & High School Curriculum Guide. Adv Sch Mental Health Promot.

[26] Jorm AF (2012). Mental health literacy: empowering the community to take action for better mental health. Am Psychol.

[27] World Health Organization (WHO) (2013). Health literacy: the solid facts.

[28] Institute of Medicine (2008). Health literacy: improving health, health systems and health policy around the world.

[29] Kickbush I (2001). Health literacy: addressing the health and education divide. Health Promot Int.

[30] Wheatley C, Beale N, Wassenaar T, Graham M, Eldridge E, Dawes H, Johansen-Berg H (2020). Fit to Study: Reflections on designing and implementing a large-scale randomized controlled trial in secondary schools. Trends Neurosci Educ.

[31] Wasserman C, Hoven CW, Wasserman D (2012). Suicide prevention for youth - a mental health awareness program: lessons learned from the saving and empowering young lives in Europe (SEYLE) intervention study. BMC Public Health.

[32] Kutcher S, Wei Y, Coniglio C (2016). Mental health literacy: past, present, and future. Can J Psychiatry.

[33] Goddard YL, Goddard RD, Bailes LP, Nichols R (2019). From school leadership to differentiated instruction. Elem Sch J.

[34] Patton MQ (2015). Qualitative evaluation and research methods.

[35] Creswell JW (2022). A concise introduction to mixed methods research.

[36] Creswell JW, Plano Clark VL (2018). Designing and conducting mixed methods research.

[37] Shenton AK (2004). Strategies for ensuring trustworthiness in qualitative research projects. Educ Inf.

[38] Charmaz K (2008). Constructionism and the grounded theory method. Handbook of constructionist research.

[39] Charmaz K (2014). Constructing grounded theory.

[40] Braun V, Clarke V (2006). Using thematic analysis in psychology. Qual Res Psychol.

[41] Lipscomb M (2012). Abductive reasoning and qualitative research. Nurs Philos.

[42] Charmaz K (1995). The body, identity, and self: adapting to impairment. Sociological Quarterly.

[43] Yamaguchi S, Foo JC, Nishida A, Ogawa S, Togo F, Sasaki T (2020). Mental health literacy programs for school teachers: A systematic review and narrative synthesis. Early Interv Psychiatry.

[44] Dunkley DM, Zurof DC, Blankstein KR (2003). Self-critical perfectionism and daily afect: dispositional and situational infuences on stress and coping. J Pers Soc Psychol.

[45] Rogers ML, Joiner TE (2017). Rumination, suicidal ideation, and suicide attempts: A meta-analytic review. Rev Gen Psychol.

[46] Shahar G (2015). Erosion: the psychopathology of self-criticism.

[47] Shahar G, Rogers ML, Shalev H, Joiner TE (2020). Selfcriticism, interpersonal conditions, and biosystemic infammation in suicidal thoughts and behaviors within mood disorders: A bio-cognitive–interpersonal hypothesis. J Pers.

[48] Calear AL, Christensen H (2010). Systematic review of school-based prevention and early intervention programs for depression. J Adolesc.

[49] Spence SH, Shortt AL (2007). Research review: can we justify the widespread dissemination of universal, school-based interventions for the prevention of depression among children and adolescents?. J Child Psychol Psychiatry.

[50] Mackenzie K, Williams C. Universal, school-based interventions to promote mental and emotional well-being: what is being done in the UK and does it work? A systematic review. BMJ Open. 2018;8(9) Available from. 10.1136/bmjopen-2018-022560. 10.1136/bmjopen-2018-022560 PMC6129100 30196267

[51] Stallard P, Buck R (2013). Preventing depression and promoting resilience: feasibility study of a school-based cognitive-behavioural intervention. Br J Psychiatry.

[52] Robinson J, Cox G, Malone A, Williamson M, Baldwin G, Fletcher K, O’Brien M (2019). A systematic review of school-based interventions aimed at preventing, treating, and responding to suicide -related behaviour in young people. Lancet Psychiatry.

[53] Durlack JA, Weissberg RP, Dymnicki AB, Taylor RD, Schellinger KB (2011). The impact of enhancing students’ social and emotional learning: A meta-analysis of school-based universal interventions. Child Dev.

[54] Lean D (2016). The status of school psychology in Ontario school boards: 2016 perspective. Can J Sch Psychol.

[55] Rodger S, Bourdage R, Hancock K, Hsiang R, Masters R, Leschied A (2016). Supporting students: A GRADE analysis of the research on student wellness and classroom mental health support. Can J Sch Psychol.

